# Development of a clinical diagnostic model for atypical endometrial hyperplasia and endometrial carcinoma in women aged 40–60 years based on refined abnormal uterine bleeding patterns

**DOI:** 10.3389/fonc.2025.1684604

**Published:** 2025-12-19

**Authors:** Shulin Huang, Xudong Hu, Lingjuan Hu, Tingting Du, Yanchun Zhao, Jing Xiao

**Affiliations:** 1 The Second Clinical College of Guangzhou University of Chinese Medicine, Guangzhou, Guangdong, China; 2 Department of Gynecology, The Second Affiliated Hospital of Guangzhou University of Chinese Medicine, Guangzhou, Guangdong, China; 3 State Key Laboratory of Traditional Chinese Medicine Syndrome/Department of Gynecology, The Second Affiliated Hospital of Guangzhou University of Chinese Medicine, Guangzhou, Guangdong, China

**Keywords:** endometrial carcinoma, atypical endometrial hyperplasia, nomogram, abnormal uterine bleeding, risk prediction model

## Abstract

**Objective:**

To develop and validate a diagnostic nomogram model for predicting atypical endometrial hyperplasia (AEH) and endometrial carcinoma (EC) in women aged 40–60 years with abnormal uterine bleeding (AUB), incorporating detailed bleeding patterns and clinical parameters.

**Methods:**

This retrospective cohort study included 1,920 patients aged 40–60 years with AUB who underwent hysteroscopic evaluation across four hospital branches from 2021 to 2024. Variables were screened via univariate logistic regression followed by LASSO regression. A multivariate logistic regression model was constructed using seven selected predictors, including age, family history of cancer, endometrial thickness, menstrual blood loss, abnormal menstrual duration, intermenstrual bleeding, and postmenopausal bleeding. Internal validation was performed using bootstrap resampling; external validation was conducted using an independent cohort.

**Results:**

The final model demonstrated good discriminatory performance (AUC = 0.814 in the training cohort, 0.762 in external validation). Calibration plots and the Hosmer–Lemeshow test indicated good agreement between predicted and observed probabilities. Decision curve analysis confirmed favorable clinical utility. Patients classified as high-risk (score ≥70.362) had a significantly increased likelihood of AEH/EC (OR = 12.46, 95% CI: 7.56–21.01, P < 0.001).

**Conclusion:**

This study presents a validated, user-friendly nomogram integrating refined AUB patterns and clinical variables to support early risk stratification for AEH/EC in women aged 40–60 years. The model demonstrates robust predictive performance and may assist in guiding individualized diagnostic strategies, particularly in resource-limited settings.

## 1 Introduction

Endometrial cancer is the second most common malignancy of the female reproductive tract and has become the most prevalent gynecologic cancer in developed countries, with its steadily increasing incidence (1).This disease poses a significant threat to women’s health. While some early-stage cases may be asymptomatic and clinically elusive, approximately 90% of patients present with abnormal vaginal bleeding as the initial symptom. Epidemiological data indicate that 10% to 15% of perimenopausal women presenting with abnormal uterine bleeding (AUB) are eventually diagnosed with endometrial cancer, for which AUB often serves as an early clinical manifestation (2).Given the rising disease burden, developing an efficient and accurate preoperative risk assessment strategy for women aged 40–60 years with AUB is of great clinical importance.

Transvaginal color Doppler ultrasound serves as a valuable adjunct in the diagnostic evaluation of gynecological diagnosis, offering advantages of convenience, non-invasiveness, and cost-effectiveness. However, its diagnostic accuracy for endometrial lesions remains highly operator-dependent. Currently, the definitive diagnosis of malignant endometrial lesions relies primarily on histopathological evaluation of endometrial tissue. Given the invasiveness of biopsy, early and noninvasive identification of high-risk individuals is essential to minimize unnecessary interventions in benign cases and improve clinical decision-making. Nomograms based on multivariate regression models been widely adopted in clinical practice for disease risk stratification and are increasingly recognized for their utility in oncologic diagnostics, prognostication, and personalized medicine (3). Previous studies have identified that various clinical variables—such as age, body mass index (BMI), family history of cancer, endometrial thickness (ET), and serum carbohydrate antigen 125 (CA125) levels—as independent predictors of endometrial carcinoma (4). However, most existing studies have focused on individual indicators or conventional clinical factors such as age, obesity, and menopausal status, while overlooking AUB patterns—specifically frequency, regularity, and duration—as potential risk factors.

Therefore, this study incorporated AUB patterns with laboratory findings and sonographic features, to systematically identify independent risk factors for endometrial atypical hyperplasia and endometrial cancer. We further assessed the diagnostic performance of each predictor and developed a clinically applicable diagnostic model based on refined bleeding patterns to facilitate early detection and precise management of premalignant endometrial lesions and endometrial carcinoma.

## 2 Materials and methods

**Figure 1:**
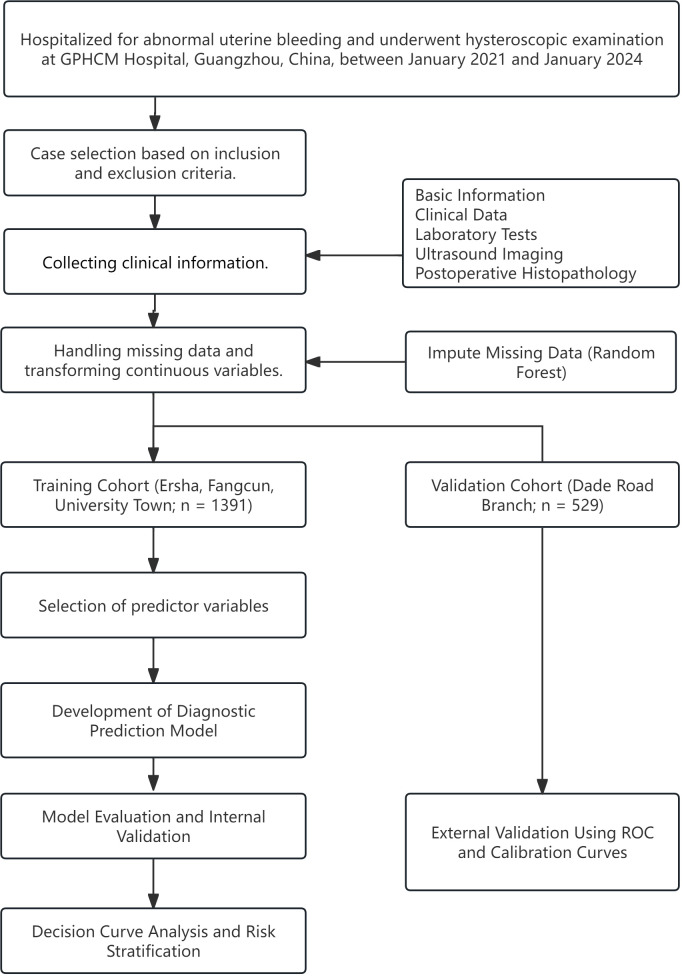
Flowchart of the study design.

This retrospective study analyzed data from patients hospitalized for AUB who underwent hysteroscopic examination between January 2021 and January 2024 at four campuses of Guangdong Provincial Hospital of Chinese Medicine (GPHCM), including the Da De Road Main Campus, University Town Hospital, Ersha Island Hospital, and Fangcun Hospital in Guangzhou, China. The study design and flow are illustrated in **Figure 1**. Given that each hospital campus operates independently with separate clinical teams and management protocols, we stratified the data by hospital. Cases from University Town, Ersha Island, and Fangcun campuses were used as the training cohort for model development, while cases from the Da De Road Main Campus served as the external validation cohort to assess the model’s applicability and generalizability. This study protocol was approved by the Ethics Committee of GPHCM (Approval to YE2025-307-01).

### 2.1 Inclusion and exclusion criteria

Diagnostic Criteria: AUB refers to bleeding from the uterine cavity that deviates from the normal menstrual cycle in terms of frequency, regularity, duration, or volume. According to the International Federation of Gynecology and Obstetrics (FIGO), AUB etiologies are classified using the PALM-COEIN system (5). The “PALM” group represents structural abnormalities identifiable via imaging or histopathology, including endometrial polyps (P), adenomyosis (A), leiomyoma (L), and malignancy or atypical hyperplasia (M). In this study, only structural causes under the PALM category were included, specifically focusing on submucosal fibroids protruding into the uterine cavity and endometrial atypical hyperplasia or carcinoma. Non-structural causes classified under the COEIN group were excluded.

Inclusion Criteria:

1. Women aged 40 to 60 years;
2. Underwent preoperative transvaginal ultrasonography;
3. Received diagnostic curettage under hysteroscopy;
4. Had Complete surgical records and confirmed histopathological results.

Exclusion Criteria:

1. Vaginal bleeding caused by lesions of the vulva, vagina, cervix, or other non-uterine reproductive tract sources;
2. Bleeding secondary to systemic coagulopathy;
3. History of other systemic malignancies;
4. Drug-induced bleeding possibly caused by medications used within the past month;
5. Incomplete or missing medical records.

### 2.2 Data collection and definition

Data were independently reviewed and recorded by two researchers in a back-to-back manner. Any discrepancies were resolved through cross-verification and adjudicated by a third researcher. The collected clinical date included:

1. Basic Information: Age, Serum CA125 level, Height, Weight, BMI, Gravidity, Parity, Number of abortions, Hypertension, Hyperlipidemia, Type 2 diabetes mellitus, Family history of cancer.
2. Menstrual History: Menarche age, Menstrual blood loss(Menstrual blood loss was evaluated using the Pictorial Blood Loss Assessment Chart (6) and categorized into three groups: Heavy menstrual bleeding, Normal menstrual bleeding, Light menstrual bleeding.), Menstrual blood color, Menopause.
3. Transvaginal Ultrasonography Findings: Overall uterine volume (calculated using the prolate ellipsoid method (7), Volume=length×depth×width×0.523), Endometrial thickness, Endometrial texture, Color doppler flow imaging(CDFI) of endometrium.
4. AUB Patterns: Data on AUB patterns were collected through a standardized clinician-administered questionnaire at the time of patient evaluation. AUB patterns were categorized based on clinical data and the FIGO PALM-COEIN classification system (**Table 1**).

**Table 1:** Definition of AUB patterns.

Categories	Pattern	Definition
Abnormal Menstrual Cycle	Early menstrual cycle	Menstrual cycle occurs ≥7 days earlier than expected.
	Delayed menstruation	Menstrual cycle is delayed ≥7 days beyond expected date.
Abnormal menstrual duration	Prolonged menstruation	Menstrual cycle exceeds 7 days with a regular cycle.
Other types	Intermenstrual bleeding	Vaginal bleeding occurs between two menstrual periods.
	Postmenopausal bleeding	Vaginal bleeding recurs ≥1 year after the onset of menopause.

### 2.3 Study grouping

Participants were classified into two groups based on the histopathological results of hysteroscopic diagnostic curettage:

Group A: Included cases with normal endometrial tissues -such as proliferative endometrium, secretory endometrium, menstrual endometrium, atrophic endometrium, and fragmented endometrial glands- endometritis, endometrial polyps, adenomyosis, submucosal uterine fibroids, and non-atypical hyperplasia.

Group B: Included cases with atypical endometrial hyperplasia (AEH) or endometrial carcinoma (EC).

### 2.4 Statistical analysis

Data were analyzed using R software (version 4.4.2) (8). Missing data constituted less than 0.1% and were imputed using the random forest algorithm in the “missForest” package. Categorical variables were presented as percentages and compared using the chi-square test. Normally distributed continuous variables were expressed as mean ± SD and compared using the independent samples t-test. Non-normally distributed variables were reported as medians (Q1, Q3) and compared using the Mann–Whitney U test.

#### 2.4.1 Variable selection and nomogram construction

In the training cohort, all variables were initially considered as potential predictors using univariate logistic regression analysis. Variables with a P-value <0.1 were then subjected to least absolute shrinkage and selection operator (LASSO) regression for feature selection. The optimal penalty parameter (λ) was determined via cross-validation based on the minimum criteria to ensure an optimal balance between model complexity and predictive performance. Subsequently, a multivariable logistic regression model was constructed to develop a diagnostic nomogram for estimating the risk of AEH/EC.

#### 2.4.2 Assessment of nomogram performance and clinical utility

The predictive performance of the nomogram was evaluated by calculating the area under the receiver operating characteristic (ROC) curve (AUC). Internal validation was conducted using the bootstrap resampling method (1,000 iterations) to estimate the 95% confidence intervals (CIs). Model calibration was assessed using the Hosmer–Lemeshow goodness-of-fit test and visualized via calibration plots. External validation was performed by applying the model to the independent validation cohort, generating ROC and calibration curves to examine generalizability. Based on the optimal cut-off value identified from the ROC analysis, patients were stratified into high- and low-risk groups. The incidence of endometrial premalignant and malignant lesions was compared between groups, and the discriminatory capability of the model in clinical stratification was further quantified using odds ratios (ORs). Finally, decision curve analysis (DCA) was conducted to evaluate the net clinical benefit and applicability of the model across a range of threshold probabilities.

## 3 Result

A total of 1,920 patients met the predefined inclusion and exclusion criteria and were enrolled in the study, with 1,391 allocated to the training cohort and 529 to the validation cohort. Baseline demographic and clinical characteristics of both cohorts are presented in **Table 2**.

**Table 2:** Baseline characteristics of the training and testing cohorts.

Variables	Total (n = 1920)	Traning cohort (n = 1391)	Validation cohort (n = 529)	*P*
Group				
Group B, n (%)	113(6)	73(5)	40(8)	0.054
Group A, n (%)	1807(94)	1318(95)	489(92)	
Age(years), Median (Q1,Q3)	46 (43, 49)	46 (43, 49)	46 (43, 49)	0.519
CA125 (U/ml)				0.666
<35	1667 (87)	1200 (86)	467 (88)	
35-100	179 (9)	134 (10)	45 (9)	
100-200	41 (2)	31 (2)	10 (2)	
>200	33 (2)	26 (2)	7 (1)	
Height(cm), Median (Q1,Q3)	158 (155, 162)	158 (155, 162)	158 (155, 162)	0.776
Weight(kg), Median (Q1,Q3)	58.2 (53, 64.8)	59 (53.5, 65)	58 (53, 64)	0.114
BMI, Median (Q1,Q3)	23.23 (21.36, 25.59)	23.31 (21.43, 25.64)	23.03 (21.23, 25.4)	0.092
Gravidity,Mean±SD	2.88±1.58	2.64±1.56	3.53±1.43	< 0.001*
Parity,Mean±SD	1.78±1.051	1.48±0.92	2.57±0.97	< 0.001*
Abortion,Mean±SD	1.38±1.27	1.16±1.26	1.97±1.10	< 0.001*
Hypertension, n (%)				0.609
No	1778 (93)	1285 (92)	493 (93)	
Yes	142 (7)	106 (8)	36 (7)	
Hyperlipidemia, n (%)				0.001*
No	1872 (98)	1346 (97)	526 (99)	
Yes	48 (2)	45 (3)	3 (1)	
Type 2 diabetes, n (%)				0.308
No	1887 (98)	1364 (98)	523 (99)	
Yes	33 (2)	27 (2)	6 (1)	
Family history of cancer, n (%)				0.929
No	1836 (96)	1331 (96)	505 (95)	
Yes	84 (4)	60 (4)	24 (5)	
Menarche age, Median (Q1,Q3)	13 (13, 14)	13 (13, 14)	14 (13, 14)	< 0.001*
Menstrual blood loss, n (%)				0.315
Heavy	350 (18)	265 (19)	85 (16)	
Moderate	1490 (78)	1068 (77)	422 (80)	
Light	80 (4)	58 (4)	22 (4)	
Menstrual blood color, n (%)				0.007*
Dark red	770 (40)	579 (42)	191 (36)	
Bright red	1140 (59)	802 (58)	338 (64)	
Pale red	10 (1)	10 (1)	0 (0)	
Abnormal menstrual cycle, n (%)				1.000
Irregular	166 (9)	120 (9)	46 (9)	
Regular	1754 (91)	1271 (91)	483 (91)	
Abnormal menstruation duration, n (%)				0.004*
Irregular	248 (13)	160 (12)	88 (17)	
Regular	1672 (87)	1231 (88)	441 (83)	
Intermenstrual bleeding, n (%)				0.681
No	1836 (96)	1328 (95)	508 (96)	
Yes	84 (4)	63 (5)	21 (4)	
postmenopausal bleeding, n (%)				< 0.001*
No	1858 (97)	1358 (98)	500 (95)	
Yes	62 (3)	33 (2)	29 (5)	
Uterine Volume, Median (Q1,Q3)	97.28 (70.6, 138.51)	97.3 (70.95, 141.44)	96.77 (69.39, 134.41)	0.325
Endometrial thickness, Median (Q1,Q3)	11 (7.18, 15)	11 (7.25, 15)	10.7 (7, 15)	0.275
Endometrial texture, n (%)				0.317
No	1883 (98)	1361 (98)	522 (99)	
Yes	37 (2)	30 (2)	7 (1)	
CDFI, n (%)				0.915
No abnormal	1698 (88)	1229 (88)	469 (89)	
Abnormal	222 (12)	162 (12)	60 (11)	

*p.value≤0.05.

### 3.1 Univariate analysis

**Figure 2:**
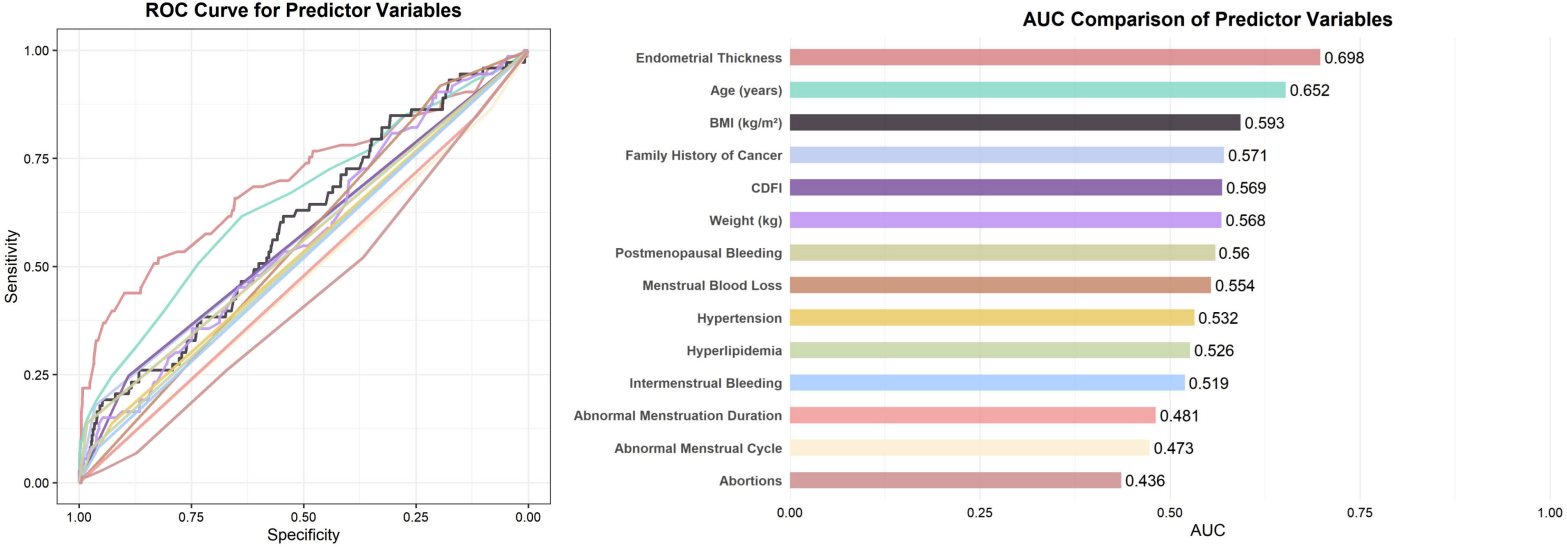
ROC curves and AUC comparison of predictor variables with p < 0.1. Note: For variables with AUC < 0.5, the association direction was opposite to the reference category (e.g., “Yes” vs. “No”); their effective discrimination can be interpreted as 1 − AUC.

To identify potential predictors associated with the outcome, all candidate variables were subjected to univariate logistic regression analysis (**Table 3**). Variables with a p-value < 0.1 were then assessed for discriminatory performance by constructing ROC curves, with corresponding AUC values calculated (**Figure 2**). The results indicated that none of the individual predictors demonstrated strong predictive ability for AEH/EC (0.4 < AUC < 0.7).

**Table 3:** Univariate logistic regression analysis of risk.

Variables	β	S.E.	Z	OR	P
Age (years)	0.167	0.032	5.236	1.182(1.11-1.26)	<0.001*
CA125 (U/ml)					
<35	-0.225	0.326	-0.689	0.799(0.44-1.58)	0.491
35-100	0.152	0.386	0.394	1.164(0.51-2.34)	0.694
100-200	0.225	0.741	0.303	1.252(0.20-4.27)	0.762
>200	0.418	0.746	0.56	1.519(0.24-5.25)	0.575
Height(cm)	-0.028	0.023	-1.224	0.972(0.93-1.02)	0.221
Weight(kg)	0.035	0.012	2.799	1.035(1.01-1.06)	0.005*
BMI	0.106	0.03	3.533	1.112(1.05-1.18)	<0.001*
Gravidity	-0.086	0.082	-1.045	0.918(0.78-1.07)	0.296
Parity	0.094	0.122	0.774	1.099(0.85-1.38)	0.439
Abortions	-0.198	0.112	-1.759	0.821(0.65-1.01)	0.079*
Hypertension (Yes vs. No)	0.703	0.357	1.973	2.021(0.95-3.90)	0.049*
Hyperlipidemia (Yes vs. No)	1.077	0.456	2.362	2.937(1.09-6.70)	0.018*
Type 2 diabetes (Yes vs. No)	-0.371	1.026	-0.361	0.69(0.04-3.32)	0.718
Family history of cancer (Yes vs. No)	1.768	0.34	5.199	5.859(2.91-11.14)	<0.001*
Menarche age	-0.103	0.086	-1.193	0.902(0.76-1.06)	0.233
Menstrual blood loss					
Heavy	-1.005	0.432	-2.327	0.366(0.14-0.79)	0.020*
Moderate	0.674	0.347	1.945	1.962(1.04-4.11)	0.052*
Light	0.305	0.533	0.573	1.357(0.40-3.43)	0.567
Menstrual blood color					
Dark red	0.154	0.242	0.637	1.166(0.72-1.87)	0.524
Bright red	-0.181	0.241	-0.751	0.834(0.52-1.34)	0.453
Pale red	0.703	1.061	0.663	2.02(0.11-10.97)	0.508
Abnormal menstrual cycle (Regular vs.Irregular)	-0.556	0.355	-1.567	0.574(0.30-1.22)	0.117
Abnormal menstruation duration (Regular vs.Irregular)	-0.331	0.339	-0.977	0.718(0.38-1.47)	0.329
Intermenstrual bleeding (Yes vs. No)	0.684	0.447	1.529	1.981(0.74-4.42)	0.126
Postmenopausal bleeding (Yes vs. No)	2.190	0.400	5.473	8.937(3.92-19.12)	<0.001*
Uterine Volume	0.001	0.001	0.624	1.001(1.00-1.00)	0.533
Endometrial thickness	0.136	0.017	7.976	1.146(1.11-1.19)	<0.001*
Endometrial texture (Homogeneous vs.Heterogeneous )	-0.482	1.023	-0.471	0.617(0.03-2.95)	0.637
CDFI (Abnormal vs. No abnormal)	0.981	0.286	3.437	2.668(1.49-4.58)	0.001*

*p value≤0.100.

### 3.2 Selection of model variables

**Figure 3:**
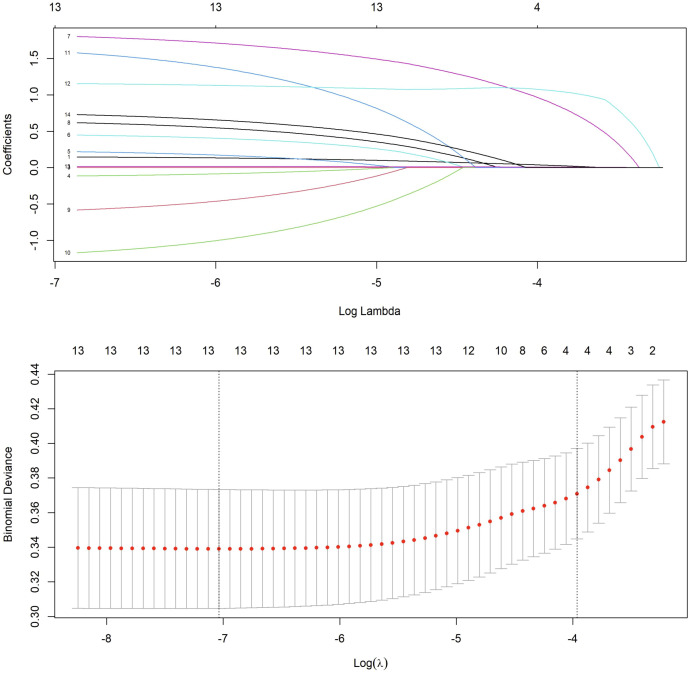
LASSO regression for feature selection with cross-validation.

Based on the univariate logistic regression analysis, variables with a p-value < 0.1 were further subjected to LASSO regression. When the penalty parameter λ = 0.0008797404, the model achieved optimal performance with minimal variable inclusion. The selected non-zero coefficient variables included age, family history of cancer, endometrial thickness, and postmenopausal bleeding (**Figure 3**). Considering clinical relevance, four additional variables-menstrual blood loss, abnormal menstrual cycle, abnormal duration of menstruation, and intermenstrual bleeding—were incorporated into the multivariable logistic regression model. The result of the multivariable analysis revealed that older age, positive family history of malignancy, increased endometrial thickness, intermenstrual bleeding, and abnormal menstrual duration were all independently associated with a higher risk of AEH/EC. In contrast, heavier menstrual bleeding was negatively associated with malignancy risk (**Table 4**).

**Table 4:** Multivariate logistic regression analysis of risk.

Variables	β	S.E.	Z	OR	P
Age	0.163	0.042	3.91	1.177(1.09–1.28)	<0.001 *
Family history of cancer					
No				Reference	
Yes	1.94	0.400	4.844	6.957(3.17–15.25)	<0.001 *
Endometrial thickness	0.149	0.019	7.946	1.161(1.12–1.20)	<0.001 *
Menstrual blood loss					
Moderate				Reference	
Heavy	-1.22	0.468	-2.607	0.295(0.12–0.74)	0.009 *
Light	0.145	0.588	0.246	1.156(0.37–3.66)	0.806
Abnormal menstruation duration					
No				Reference	
Yes	1.384	0.389	-3.562	3.992(1.86–8.55)	<0.001 *
Intermenstrual bleeding					
No				Reference	
Yes	1.827	0.549	3.331	6.218(2.12–18.23)	<0.001 *
Postmenopausal bleeding					
No				Reference	
Yes	1.083	0.580	1.867	2.953(0.95–9.20)	0.062
Abnormal menstrual cycle					
No				Reference	
Yes	-0.548	0.432	-1.269	0.578(0.25–1.35)	0.204

*p value≤0 05.

### 3.3 Construction and validation of the predictive model

**Figure 4:**
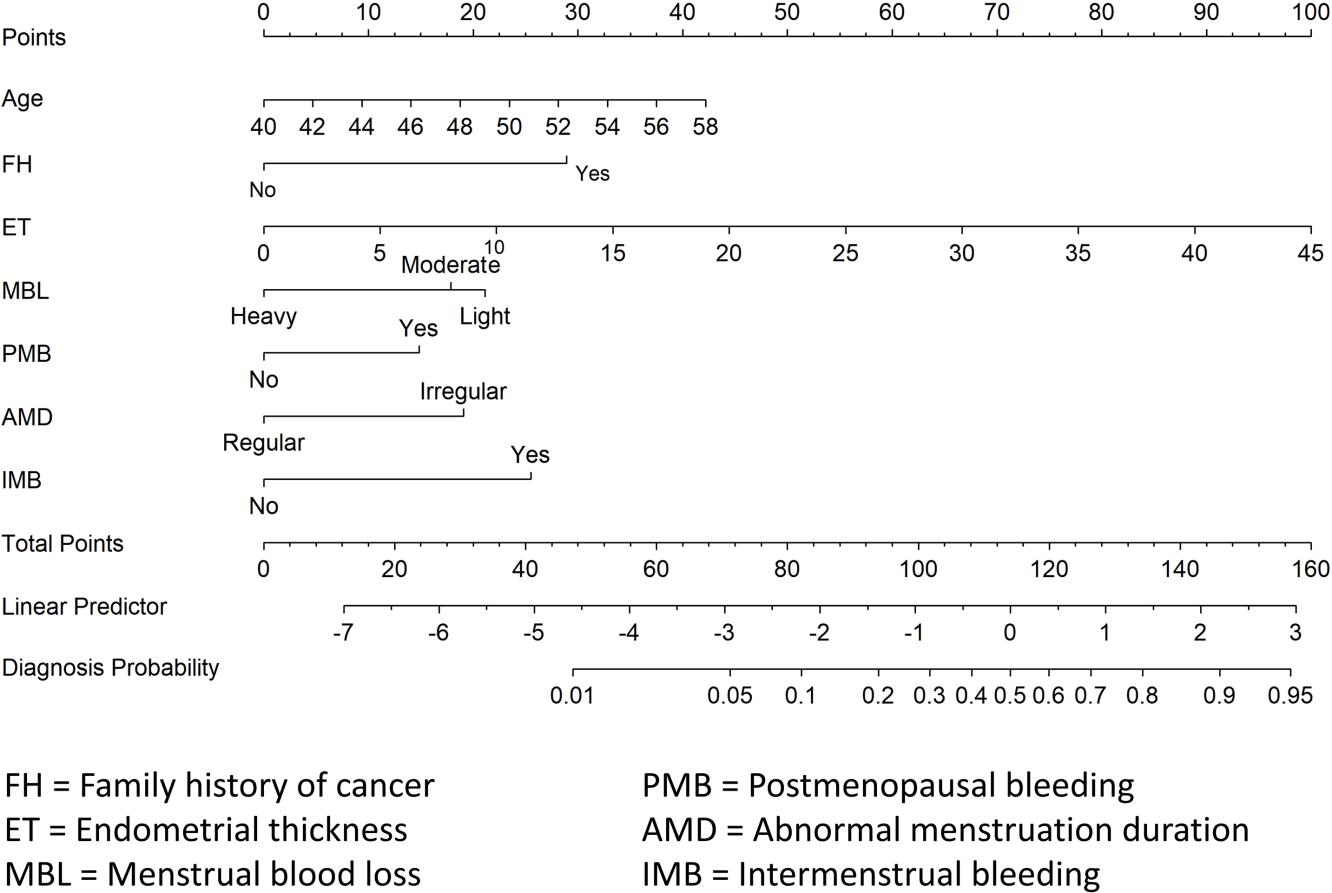
Diagnostic nomogram for predicting the risk of AEH/EC.

**Figure 5:**
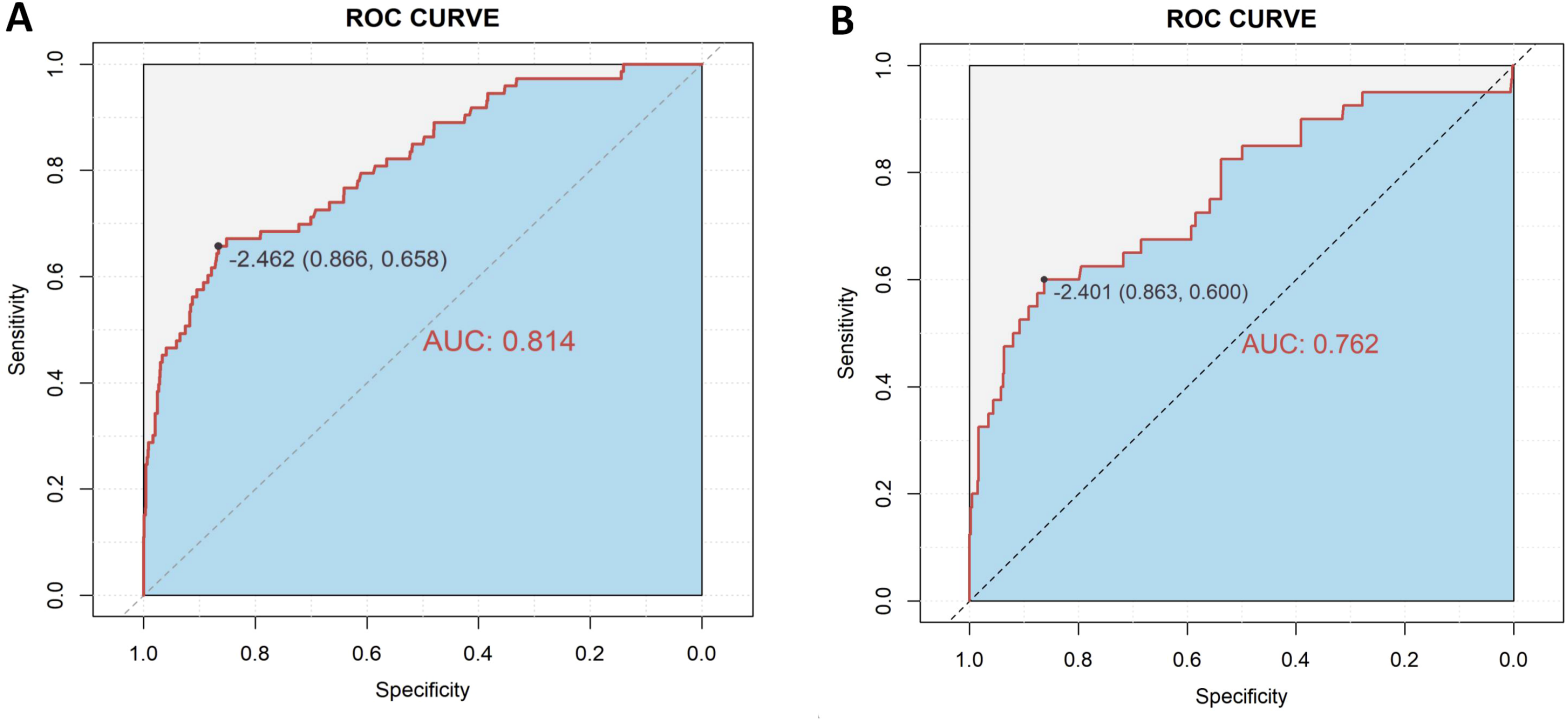
ROC curves for model performance in the training **(A)** and validation **(B)** cohorts.

**Figure 6:**
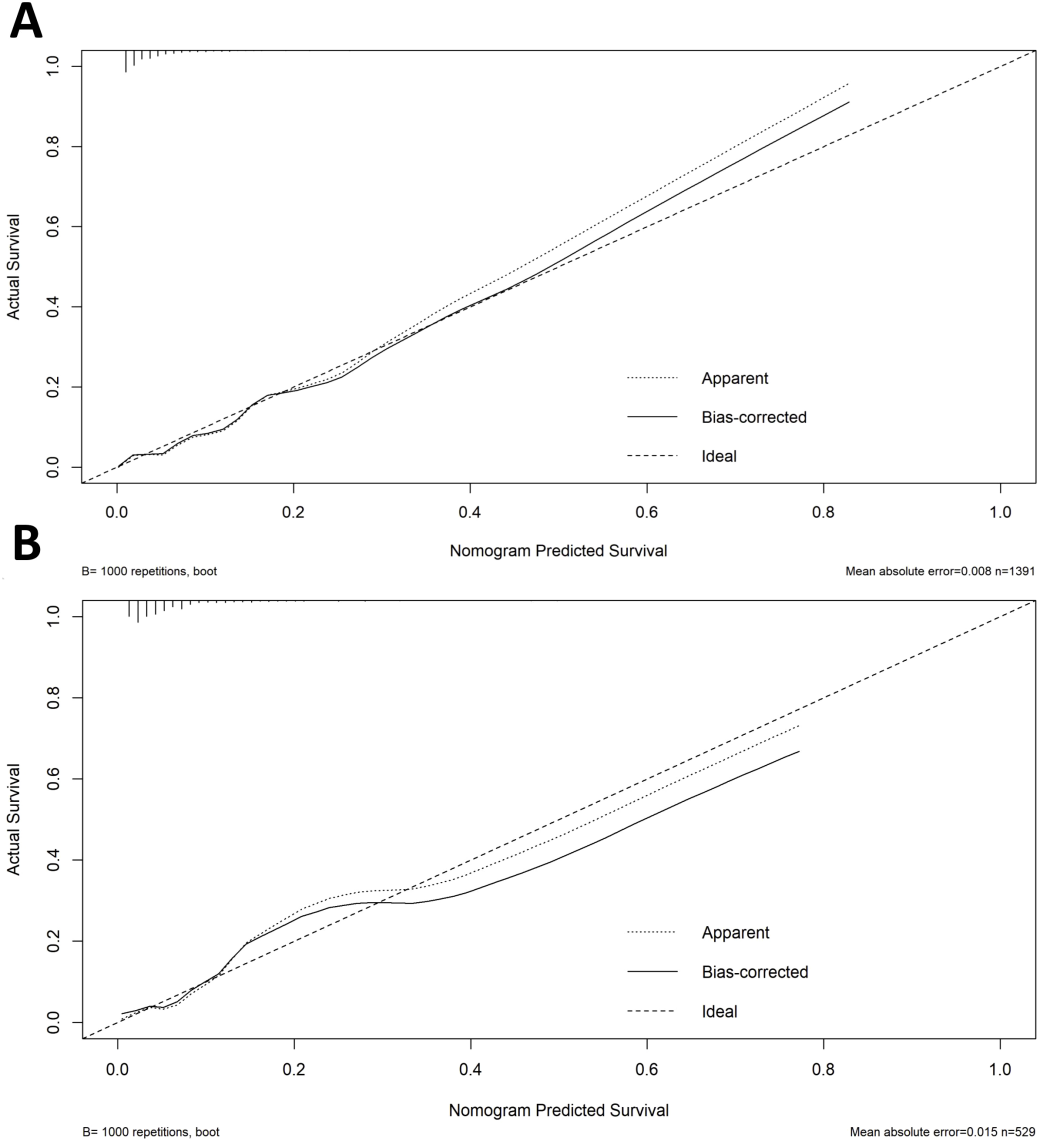
Calibration curves for nomogram prediction in the training **(A)** and validation **(B)** cohorts.

A diagnostic nomogram was constructed to estimate the risk of AEH/EC based on seven variables: age, family history of cancer, endometrial thickness, menstrual blood loss, abnormal menstrual duration, intermenstrual bleeding, and postmenopausal bleeding (**Figure 4**). The model demonstrated favorable discriminatory performance, with an AUC of 0.814 in the training cohort (**Figure 5A**). Internal validation using bootstrap resampling (1,000 iterations) yielded a 95% CI for the AUC of 0.760 to 0.867. The Hosmer–Lemeshow goodness-of-fit test (P = 0.1649) and the calibration plot (**Figure 6A**) indicated satisfactory agreement between predicted and observed outcomes in the training cohort. External validation using the independent validation cohort produced an AUC of 0.762 (95% CI: 0.671–0.851)(**Figure 5B**), and the corresponding calibration curve (**Figure 6B**) further confirmed the model’s stability and predictive accuracy across datasets.

### 3.4 Risk stratification

To evaluate the clinical stratification capacity of the nomogram, total scores were calculated for each patient based on the established multivariable logistic regression model. The optimal cut-off value was determined using the Youden index derived from the ROC curve (**Figure 5**), yielding a score threshold of 70.362, which corresponded to a logit value of –2.462. Patients were stratified into high-risk (score ≥70.362) and low-risk (score <70.362) groups accordingly. Logistic regression analysis revealed a significantly higher incidence of AEH/EC in the high-risk group compared to the low-risk group, with an OR of 12.46 (95% CI: 7.56–21.01, P < 0.001).

### 3.5 Clinical utility analysis

**Figure 7:**
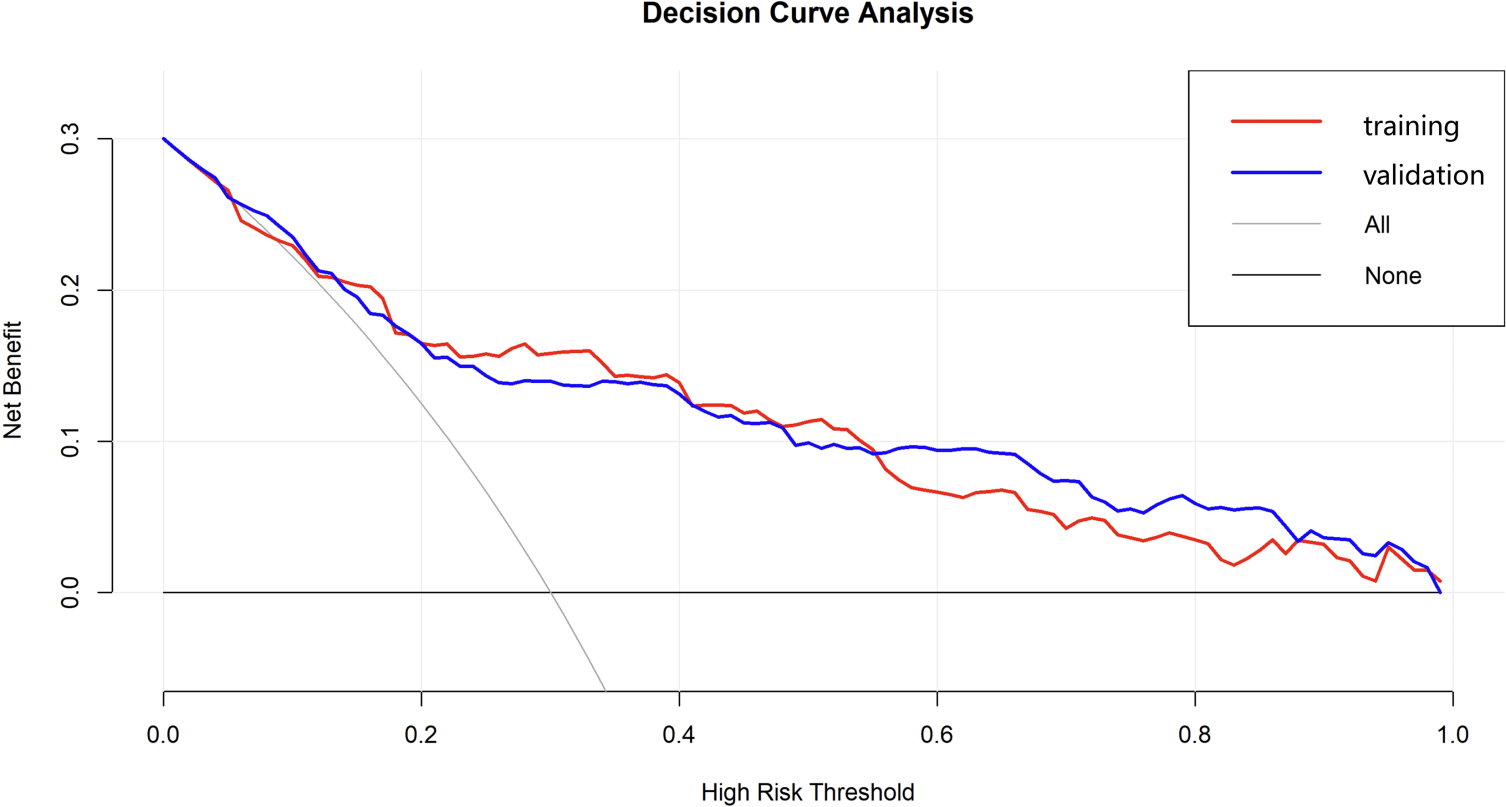
Decision curve analysis of the nomogram model for predicting AEH/EC.

The DCA was performed to evaluate the clinical applicability of the nomogram model for predicting AEH/EC (**Figure 7**). The DCA curve consistently demonstrated a higher net clinical benefit across a range of threshold probabilities compared to the two default strategies-”treat-all” and “treat-none”-indicating favorable clinical utility. In the external validation cohort, the DCA results showed similarly stable performance, supporting the robustness and generalizability of the nomogram across diverse patient populations.

## 4 Discussion

In recent years, the incidence of EC has steadily increased (9). In clinical practice, AUB-particularly among perimenopausal and postmenopausal women-should raise significant concern for potential precancerous or malignant endometrial lesions (10). To addressing this clinical concern, the present study focused on perimenopausal patients presenting with AUB and systematically collected a comprehensive set of clinical characteristics. Using both univariate and multivariable logistic regression analyses, five independent risk factors were identified: advanced age, family history of malignancy, increased endometrial thickness, moderate menstrual blood loss, and intermenstrual bleeding. Notably, this study is among the first to incorporate AUB patterns as a core predictive variable alongside conventional clinical parameters in constructing a risk prediction model. The nomogram demonstrated favorable discrimination, calibration, and clinical utility in both the training and external validation cohorts. These findings indicate that the potential of mode as a practical and accessible tool for preoperative risk stratification and clinical decision-making in women with AUB.

Numerous studies have established that the development of malignant endometrial lesions is influenced by a variety of clinical risk factors. Conventional predictors—such as advanced age, positive family history of malignancy, increased EMT, and postmenopausal bleeding—have been widely incorporated into predictive models for risk stratification. Consistent with prior literature, the present study identified age, family history of cancer, EMT, and postmenopausal bleeding as independent predictors of AEH/EC (11). Burbos et al. (12)conducted a multivariable analysis of 3,047 women with postmenopausal bleeding and identified diabetes, EMT ≥14 mm, recurrent bleeding episodes, age ≥64 years, and BMI ≥31 kg/m² as independent predictors of EC, developing the DEFAB risk model. In another retrospective cross-sectional study involving 212 postmenopausal women, Sebastian et al. (13)demonstrated that age ≥55 years, frequent bleeding episodes (≥2), diabetes, and endometrial thickening were independent risk factors for EC. Wynants et al. (14)constructed a multivariable prediction model in 2,417 women undergoing ultrasonography for AUB, using age, BMI, and sonographic features as core predictors. However, the aforementioned studies have not focused on the predictive value of specific AUB patterns in endometrial malignancy. Jha et al. (15) reported a potential association between intermenstrual bleeding (IMB) and AEH/EC, although detailed bleeding patterns were not incorporated into their predictive model. Similarly, in the predictive model developed by Burbos et al. (16), while data on vaginal bleeding volume and duration were recorded, these parameters were not found to be independent predictors of malignancy—potentially due to imprecise definitions of bleeding types or population heterogeneity. In our model, endometrial thickness (ET) emerged as the most powerful single predictor of AEH/EC, consistent with previous findings. Nevertheless, integrating refined AUB patterns and demographic factors yielded superior predictive performance compared with ET alone (AUC 0.814 vs. 0.698), while maintaining moderate discrimination in the external validation cohort (AUC 0.762). This suggests that while ET predominantly drives model discrimination, the integrated model combining AUB patterns with other clinical variables further improves diagnostic specificity by identifying benign thickened endometria with low malignant potential.

Our study identified IMB as an independent risk factor for AEH/EC in women aged 40–60 years with AUB, offering novel evidence for its diagnostic relevance. The pathogenesis of EC is intricately linked to prolonged estrogen exposure unopposed by progesterone. Histologically, both AEH and EC are frequently characterized by atypical glandular structures and neovascularization (17), which can compromise endometrial integrity and result in irregular or unpredictable bleeding, typically manifesting as IMB. Furthermore, hyper-estrogenic states can cause endometrial thickening, menstrual cycle irregularity, and vascular fragility, all of which contribute to IMB (18). The cyclical imbalance in endometrial shedding and repair, combined with local vascular malformations or tumor-induced angiogenesis, may underlie the clinical presentation of IMB in these patients. Given these mechanistic links, persistent or recurrent IMB, especially in older or high-risk individuals (19), should prompt evaluation to exclude underlying malignancy.

Interestingly, our analysis revealed an inverse association between heavy menstrual bleeding and malignant pathology, which may appear counterintuitive. However, this observation could be explained by several underlying clinical mechanisms. Heavy menstrual bleeding is frequently linked to benign gynecologic conditions such as submucosal fibroids, endometrial polyps, and adenomyosis, all of which are associated with increased menstrual volume. Moreover, regular heavy bleeding may indicate ovulatory cycles and sufficient progesterone production (20), offering protective effects against unopposed estrogen stimulation. Nevertheless, this association warrants cautious interpretation, as heavy menstrual bleeding may also occur in cases of endometrial hyperplasia or early EC.

Nomograms are widely accepted tools in oncology for risk prediction, integrating multiple clinical variables into a personalized risk score that is easily interpretable and clinically applicable (21). In this study, we developed a novel nomogram model to predict the risk of AEH/EC in women aged 40–60 years with AUB. Compared with conventional modeling strategies, our study introduced the LASSO regression based on logistic regression, which not only effectively identified the most relevant predictors but also addressed multicollinearity and simplified the model, thereby improving generalizability and reducing the risk of overfitting. The model exhibited favorable discrimination and calibration performance in both the training and external validation cohorts. Furthermore, DCA revealed stable net clinical benefit across a broad spectrum of threshold probabilities, supporting the model’s clinical utility. Risk stratification using the optimal cutoff value derived from the ROC curve (70.362) demonstrated that patients in the high-risk group had a 12.46-fold increased likelihood of developing AEH/EC compared to those in the low-risk group (OR = 12.46; 95% CI: 7.56–21.01; P < 0.001), indicating a strong discriminatory capacity. Compared with previously published models for predicting endometrial malignancies (22, 23), our nomogram incorporates symptom-based predictors and validates their independent prognostic value, whose accessibility, simplicity, and robust performance underscore the potential for broad clinical implementation, especially in resource-limited or primary care settings.

Despite the favorable performance of our model, several limitations must be noted. First, the study adopted a retrospective design, and the majority of clinical data were extracted from hospital information systems. Certain variables were based on patient self-reports, which may have introduced recall bias. Second, molecular biomarkers and genomic features were not included in the current model. Future research integrating molecular subtyping and genomic profiling may improve the precision and clinical utility of nomogram. Third, The diagnostic criteria for AUB differ between premenopausal and postmenopausal women. As this study focused on predicting AEH/EC in a transitional population without explicitly separating these physiological stages, such differences may affect the model’s generalizability. Future studies with larger and stratified cohorts are needed to validate the model separately in well-defined premenopausal and postmenopausal subgroups. Fourth, information on prior progestin therapy was not available in our dataset. Because progestins can affect endometrial morphology and related clinical indicators, the lack of these data may influence model accuracy. Future studies incorporating progestin exposure are warranted to further improve predictive performance.

## 5 Conclusion

This study developed a novel and clinically applicable nomogram model for predicting AEH/EC in women aged 40–60 years presenting with AUB. The model demonstrated strong predictive performance and calibration and provides a convenient and evidence-based method for preoperative risk stratification. It may serve as a valuable tool to facilitate the early identification of high-risk individuals and support personalized clinical management strategies.

## Data Availability

The raw data supporting the conclusions of this article will be made available by the authors, without undue reservation.
